# Intermittent exogenous ketosis does not reduce acute mountain sickness during early terrestrial high-altitude acclimatization

**DOI:** 10.1007/s00421-026-06426-z

**Published:** 2026-09-08

**Authors:** Domen Tominec, Myrthe Stalmans, Benjamin J. Narang, Vincent Pialoux, Grégoire P. Millet, Chiel Poffé, Tadej Debevec

**Affiliations:** 1 Faculty of Sport, University of Ljubljana, Ljubljana, Slovenia; 2 Exercise Physiology Research Group, Department of Movement Sciences, KU Leuven, Heverlee, Belgium; 3 Department of Cellular & Physiological Sciences, University of British Columbia, Vancouver, BC Canada; 4 Department of Automatics, Biocybernetics and Robotics, Jožef Stefan Institute, Ljubljana, Slovenia; 5 Université Claude Bernard Lyon 1, Laboratoire Interuniversitaire de Biologie de La Motricité UR 7424, Lyon, France; 6 Institute of Sport Sciences, University of Lausanne, Écublens, Switzerland; 7 REVAL—Rehabilitation Research Center, Faculty of Rehabilitation Sciences, Hasselt University, Diepenbeek, Belgium

**Keywords:** Acute mountain sickness, Altitude acclimatization, Hypoxia, Ketones

## Abstract

**Purpose:**

Intermittent exogenous ketosis via ketone monoester (KE) ingestion has been shown to enhance physiological responses and reduce acute mountain sickness (AMS) symptoms during acute normobaric hypoxia. Its effects during early acclimatization to terrestrial high-altitude, however, remain unclear.

**Methods:**

Thirty-four participants were randomised to an intermittent exogenous ketosis (IEK, *n*=17) or placebo (PLA, *n*=17) group and underwent a near sea level trial (295 m) without supplementation, followed by a four-day sojourn at 3375 m during which they received regular KE (IEK) or placebo (PLA) supplements. AMS symptoms, hematological markers, and resting ventilatory, cardiovascular, and cerebral tissue oxygenation responses were assessed each day upon waking, at midday, and before sleep throughout the exposure.

**Results:**

KE consistently elevated circulating β-hydroxybutyrate post-ingestion (*P*<0.001), confirming intermittent exogenous ketosis in the IEK group. AMS incidence peaked at 25–35% after 8 h, however incidence and symptom severity were similar in IEK *vs*. PLA across the altitude exposure (*P*≥0.822). KE ingestion induced (keto)acidosis, resulting in augmented ventilatory response in IEK (*P*≤0.037), but failed to mitigate hypoxia-induced blood and cerebral deoxygenation relative to PLA (*P*≥0.234). KE ingestion increased daytime cardiac output and lowered morning blood pressure in IEK (*P*≤0.031), while it did not affect autonomic, eryrthropoietic, or redox responses compared to PLA (*P*≥0.099).

**Conclusion:**

Although KE alters some physiological responses, it does not seem to importantly modulate AMS incidence and severity during early terrestrial altitude acclimatization.

**Graphical Abstract:**

**Figure:**
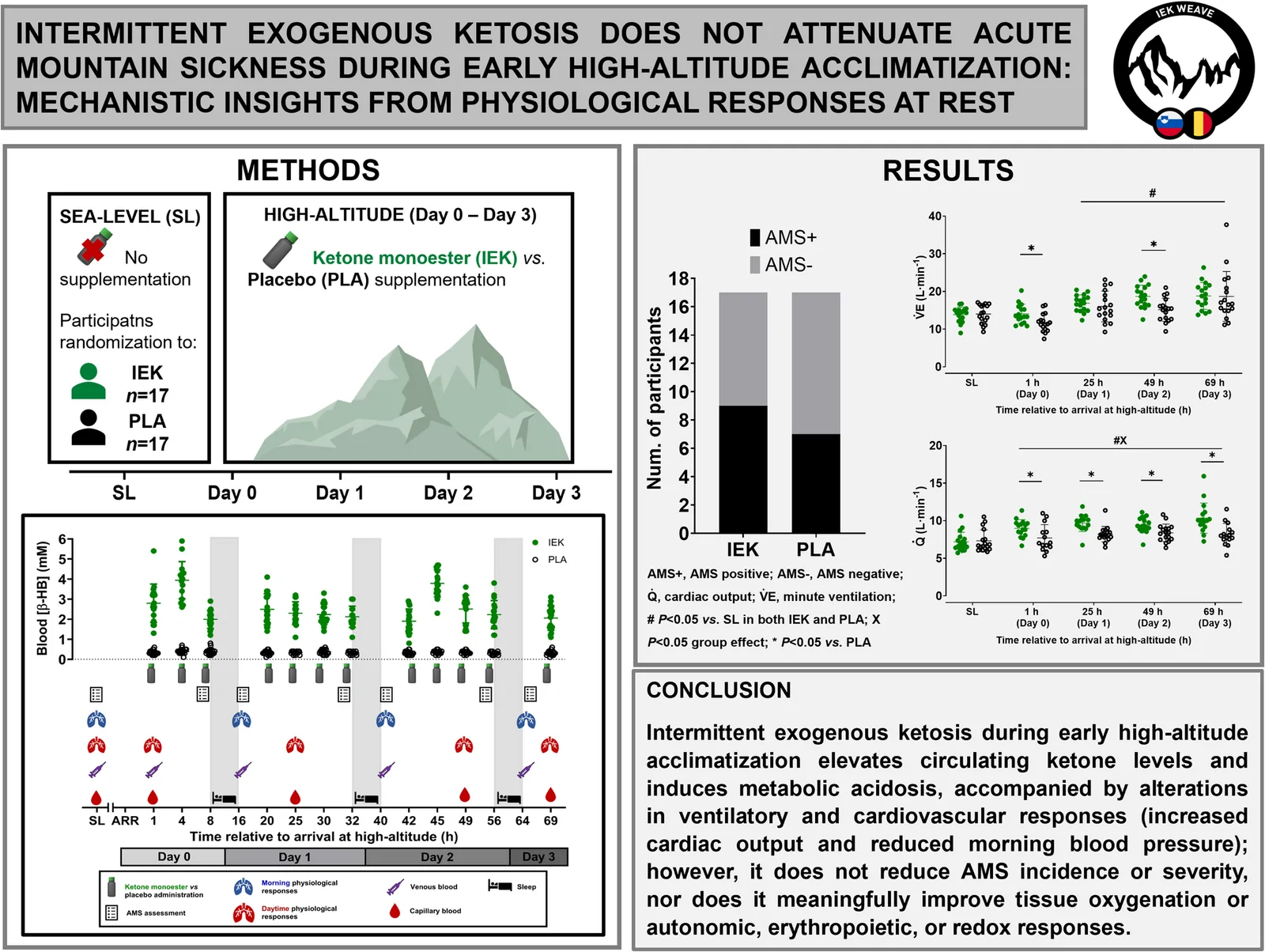

## Introduction

High-altitude-related reductions in ambient air pressure (*i.e.* hypobaria) provoke systemic hypoxemia. In response, a number of rapid compensatory responses are initiated within the first few minutes to days at high-altitude, including increases in ventilation (V̇E), heightened sympathetic neural activity, and reductions in plasma volume (Mallet et al. 2021, 2023). However, despite these physiological adjustments, acute mountain sickness (AMS) is often observed at high-altitude (Burtscher et al. 2021; Berger et al. 2020). The incidence of AMS increases progressively above ~2500 m, with symptom severity increasing in parallel with further increases in altitude (Beidleman et al. 2013; Burtscher et al. 2021). AMS is characterized by a number of nonspecific symptoms, including headache, nausea, vomiting, fatigue, and dizziness, which substantially impair functional capacity and health (Roach et al. 2018).

A reduction in blood oxygen saturation (SpO_2_) is associated with an increased risk of AMS and may predict AMS susceptibility (Burtscher et al. 2004). However, several related pathophysiological mechanisms are likely associated with AMS development, including inadequate ventilatory acclimatization resulting in hypoventilation, altered autonomic balance and increased cellular oxygen utilization (Berger et al. 2020), as well as elevations in oxidative stress accompanied by reduced antioxidant activity (Pena et al. 2022).

Recent work has demonstrated that increasing blood ketone bodies via ketone monoester (KE) administration (*i.e.* exogenous ketosis) may increase hypoxic tolerance (Stalmans et al. 2024). Indeed, findings from our lab and others suggest that KE ingestion enhances the V̇E response and attenuates the decline in SpO_2_ during both rest and submaximal exercise in normobaric hypoxia (Poffe et al. 2021a; Stalmans et al. 2024; Stalmans et al. 2025a; Stalmans et al. 2025b; McClure et al. 2024a; McClure et al. 2024b). This attenuated hypoxemia most likely underly the reductions in AMS symptoms during (acute) simulated altitude exposure (~ 4000 m) (Stalmans et al. 2024). In addition, KE ingestion under acute normobaric hypoxia has been shown to enhance cerebral oxygenation (Stalmans et al. 2024; Stalmans et al. 2025a; Vermaerke et al. 2026), as well as to increase resting heart rate (HR) and sympathetic activation (Stalmans et al. 2024; Stalmans et al. 2025). These effects have been partially corroborated in terrestrial high-altitude environments (3375 m) after two days of exposure (Narang et al. 2025). Moreover, acute exogenous ketosis has been associated with an augmented cardiac output (Q̇) in normoxia (Selvaraj et al. 2022; Nielsen et al. 2019; Gopalasingam et al. 2024).

Beyond its effects on integrative oxygen delivery, ketone bodies also exert diverse signaling functions and can thereby potentially facilitate high-altitude acclimatization (Robberechts and Poffe 2023). In particular, KE administration may support hypoxia-induced hematological responses, as evidenced by augmented serum erythropoietin concentration ([EPO]) following both acute post-exercise ketosis (Evans et al. 2023), and after daily supplementation during a 3-week overreaching training period (Poffe et al. 2023). Furthermore, ketone bodies appear to attenuate serum growth differentiation factor 15 ([GDF15]) secretion (Poffé et al. 2020; Poffe et al. 2021a), as well as reduce oxidative stress (Walsh et al. 2021) through the upregulation of enzymes involved in antioxidant and anti-inflammatory pathways (Robberechts and Poffe 2023).

These findings provide compelling evidence that KE administration might improve hypoxic tolerance, by mitigating AMS symptoms and facilitating physiological acclimatization. However, the majority of studies have been conducted using normobaric hypoxia (~ 2500–6100 m) and relatively short exposure durations (≤ 29 h). Evidence suggests that, for an equivalent hypoxic dose, hypobaric hypoxia elicits greater AMS severity and incidence (DiPasquale et al. 2016), increased alveolar dead space (Savourey et al. 2003; Faiss et al. 2013) resulting in greater systemic desaturation (Savourey et al. 2003) and provokes exaggerated oxidative stress levels (Ribon et al. 2016; Faiss et al. 2013) together with blunted antioxidant capacity (Faiss et al. 2013), when compared with a normobaric equivalent stimulus. Acclimatization in normobaric hypoxia also appears to be less effective than at terrestrial altitude (Millet et al. 2013; Fulco et al. 2013). Importantly, it remains unclear whether the effects of KE are consistent across different hypoxic doses and between simulated laboratory and terrestrial field high-altitude settings, inherently associated with greater environmental and behavioural variability (e.g., dry air, low temperatures, malnutrition). Therefore, investigating whether the observed KE-induced effects in normobaric hypoxia can be extrapolated to terrestrial high-altitude across different hypoxic levels and prolonged exposures is warranted. Given the multifactorial nature of AMS, assessment of associated physiological responses may also provide further mechanistic insight into the potential effects of exogenous ketosis.

Accordingly, we aimed to identify the effects of exogenous ketosis on AMS development as well as the associated responses of the ventilatory, cardiovascular, and cerebral systems, together with selected haematological markers during early high-altitude acclimatization*.* Our primary hypothesis was that exogenous ketosis would reduce AMS incidence and symptom severity across a four-day sojourn at 3375 m. We further hypothesised that these differences would be underpinned by attenuated SpO_2_ reductions, enhanced cerebral oxygenation, elevated [EPO] and altered redox balance and autonomic modulation.

## Methods

### Participants and ethical approval

The study received approval from the National Committee for Medical Ethics at the Ministry of Health of the Republic of Slovenia (No. 0120–524/2022/3) and the Aosta Hospital Ethical Committee (06/05/2021.0038781.I). It was preregistered at ClinicalTrials.gov (NCT06097754) and conducted in accordance with the principles of the Declaration of Helsinki. A total of 34 participants (28 males and 6 females) were recruited through an open enrolment procedure and successfully completed the study. Eligibility criteria included age between 18 and 35 years, a body mass index (BMI) of 18.5–25.0 kg⋅m^−2^, recreational physical activity corresponding to at least Tier 1 classification (McKay et al. 2022), and permanent residence <1000 m. Exclusion criteria comprised of smoking, a history of chronic diseases, regular use of medication or exogenous ketone supplements, adherence to a ketogenic diet, and exposure to altitudes ≥2000 m within one month prior to each trial. Written informed consent was obtained from all participants, who underwent medical screening before inclusion. Participants were instructed to maintain their habitual physical activity levels and dietary practices throughout the entire study.

### Study design and ascent profile

**Fig. 1:**
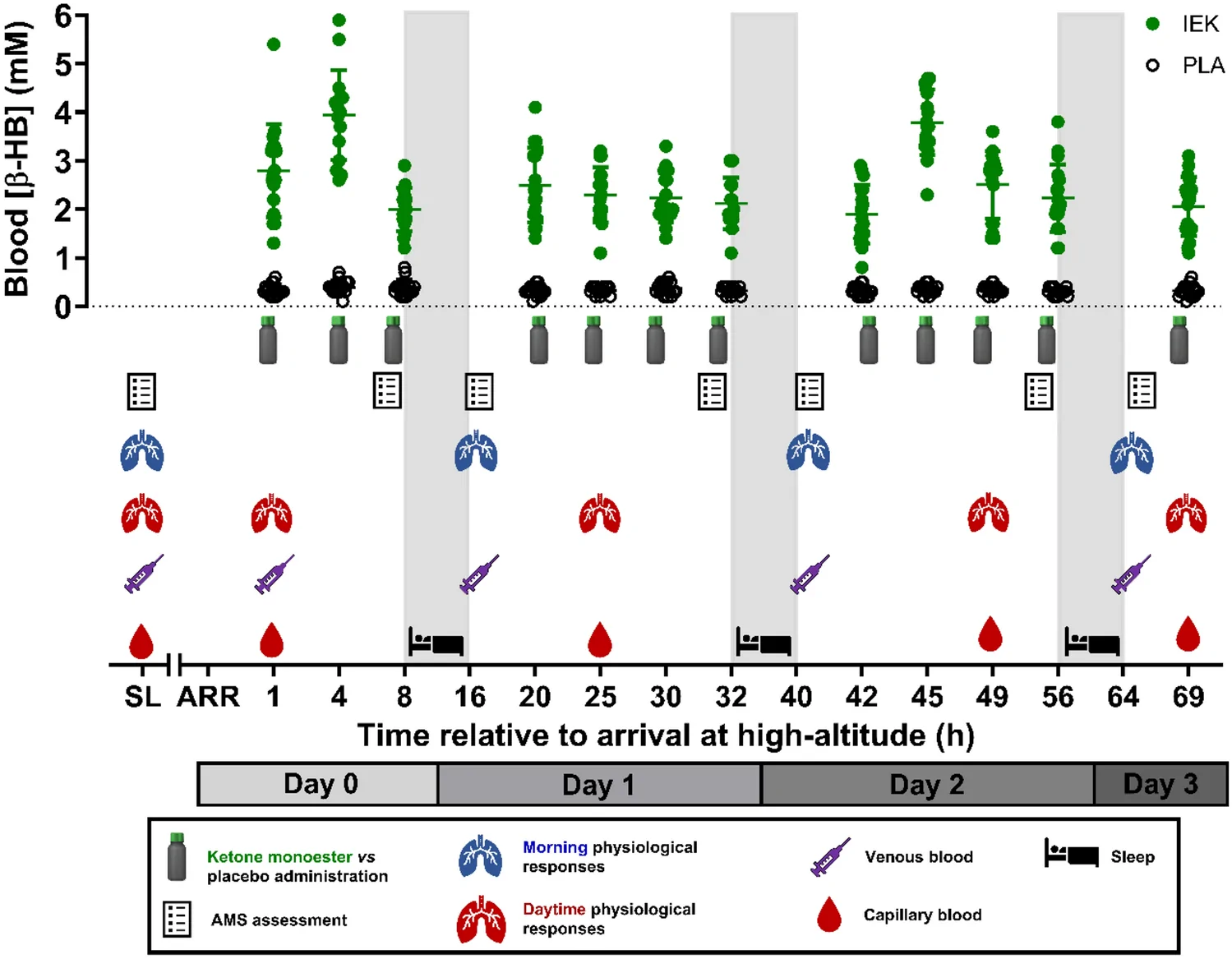
Schematic representation of the experimental protocol, depicting the supplementation schedule and the timing of both resting measurements and blood sampling across four days of high-altitude sojourn at 3375 m in participants assigned to the intermittent exogenous ketosis (IEK; green) and placebo (PLA; black) groups. Data are shown as individual values (solid and open circles) with group means (horizontal lines) ± SD (whiskers). Supplements were administered 30 min before daytime measurements and sleep, with blood β-hydroxybutyrate concentrations ([β-HB]) then measured at the onset of measurements/sleep. Grey shaded areas denote periods of sleep. *AMS* acute mountain sickness, *ARR* arrival, *SL* sea level

This study employed a randomized, double-blind, placebo-controlled, parallel-group design and was conducted as part of a broader international research project the methodological and contextual details of which have been reported previously (Narang et al. 2025; Tominec et al. 2025, 2026). The present analysis, schematically illustrated in Fig. 1, specifically examines AMS together with resting physiological data upon waking and in the early afternoon (henceforth referred to as ‘daytime’ measurements). Participants were randomly allocated to either the intermittent exogenous ketosis (IEK, *n*=17; 3 females) or placebo group (PLA, *n*=17; 3 females). All participants underwent a near sea level trial (SL) in the laboratory facilities in Ljubljana, Slovenia (altitude: 295 m; barometric pressure: 735 ± 4 mmHg; ambient temperature: 26 ± 1 °C; relative humidity: 52 ± 5%), during which no supplements were administered to establish a true normoxic baseline. After 44 ± 15 days, participants underwent a four-day (70 ± 1 h) high-altitude sojourn at Rifugio Torino, Mont Blanc (altitude: 3375 m; barometric pressure: 509 ± 7 mmHg; ambient temperature: 23 ± 2 °C; relative humidity: 36 ± 6%) with regular KE (IEK group) or placebo (PLA group) administration. Participants reached the Torino Hut at (hh:mm) 14:00 ± 00:36 via cable car ascent (20 min) from Courmayeur (1224 m), following an ~8 h drive from Ljubljana.

### Nutritional intervention

To intermittently induce exogenous ketosis throughout the four-day high-altitude sojourn, participants in the IEK group ingested daily doses of the KE (*R*)− 3-hydroxybutyl (*R*)− 3-hydroxybutyrate (KetoneAid Inc., Falls Church, Virginia, USA; ~7 kcal/g). These KE supplements were administered in 25 g boluses 30 min prior to experimental sessions and sleep. This regimen yielded a cumulative KE intake of 300 g (4.32 ± 0.64 g⋅kg^−1^ body mass; ~2100 kcal) over 69 h of exposure at 3375 m. At the same time points, participants in the PLA group consumed an equivalent volume of a non-isocaloric, taste- and viscosity-matched placebo (4.40 ± 0.60 g⋅kg^−1^ body mass; 0 kcal), consisting of 1 mM bitter sucrose octaacetate (Sigma-Aldrich, Bornem, Belgium) dissolved in water. To further mask the supplements, both KE and placebo were supplemented with 5% w/v sucralose (MyProtein, New York City, USA) and 1.0% v/v strawberry flavor drops (MyProtein, New York City, USA). All supplements were administered in opaque 50 mL tubes and immediately followed by ingestion of a sugar- and caffeine-free beverage (The Coca-Cola Company, Atlanta, Georgia, USA), thereby minimizing the likelihood of visual or gustatory identification. All supplement administration was performed by an independent researcher not otherwise involved in the study. To evaluate blinding success, participants were asked at the end of the high-altitude sojourn to indicate their perceived group assignment and to rate their confidence on a continuous scale (0%=no idea; 100%=completely certain).

30 min following each supplementation timepoint, capillary blood samples were collected from hyperemic earlobe for immediate determination of β-hydroxybutyrate ([β-HB]) using a handheld monitor (GlucoMen Areo 2 K, equipped with β-ketone sensor strips; A. Menarini Diagnostics, Florence, Italy). To facilitate double-blinding, these measurements were performed by an investigator who was otherwise not involved in the experiments.

### Acute mountain sickness symptoms

To assess AMS incidence and severity, participants filled in the initial four self-reported components of the 2018 Lake Louise Acute Mountain Sickness Score (Headache; Gastrointestinal symptoms; Fatigue and/or Weakness; Dizziness/Light-headedness) (Roach et al. 2018) upon awakening in the morning and/or before sleep in the night at SL and high-altitude (8 h, 16 h, 32 h, 40 h, 56 h, and 64 h post-arrival). AMS incidence was determined following the official guidelines of the Lake Louise scoring system, where a positive diagnosis required a headache score of ≥1 combined with a total score of ≥3.

### Morning physiological responses

Select cardiorespiratory variables were measured immediately upon waking once at SL and on the first (~ 16 h), second (~ 40 h), and third (~ 64 h) mornings of the high-altitude exposure. Upon waking, participants first used the toilet to empty their bladder and subsequently returned directly to bed. They were then instructed to remain supine with their eyes closed for an initial 10-min stabilization period, during which all measurement equipment was fitted. This was followed by a 10-min measurement period, during which heart rate (HR) was continuously recorded using a chest strap (Polar H9, Polar Electro, Kempele, Finland) and the Elite HRV mobile application (ELITE HRV, INC., Austin, USA). R–R intervals were exported and analysed with the RHRV package (version 4.4.2, R Core Team, Vienna, Austria) to derive heart rate variability (HRV) parameters, whereas the mean HR during the final 60 s of the recording was used for subsequent analyses. SpO_2_ was monitored simultaneously using a pulse oximeter (Nonin WristOx2 3150, Plymouth, MN, USA), with the average displayed value visually recorded by the researcher during the final 60 s of the recording. Participants then moved to a semirecumbent position so systolic (SBP) and diastolic (DBP) blood pressure could be measured using an automated sphygmonamometer (Omron M4, Omron Healthcare, Kyoto, Japan). SBP and DBP were used to calculate mean arterial pressure: MAP = DBP + 1/3 × (SBP – DBP).

### Daytime physiological responses

For the daytime measurements, cardiorespiratory variables were evaluated in an upright seated position on a cycle ergometer during a pre-exercise resting period at SL, upon arrival (~ 1 h), and on the afternoons of the first (~ 25 h), second (~ 49 h), and third (~ 69 h) days of high-altitude exposure. Detailed explanations of device preparation, data sampling and processing have been reported previously (Tominec et al. 2025, 2026). Cerebral tissue saturation index (Brain TSI) was assessed using a continuous-wave near-infrared spectroscopy (NIRS) device (PortaLite, Artinis Medical Systems, Elst, The Netherlands). Following standard skin preparation procedures, the cerebral NIRS sensor was positioned over the left prefrontal cortex, with sensor placement standardized across all testing sessions. Ventilatory and gas-exchange indices, including oxygen uptake(V̇O_2_), minute V̇E, and end-tidal partial pressure of carbon dioxide (P_ET_CO_2_), were measured breath-by-breath using a metabolic cart (Quark CPET, COSMED, Rome, Italy). Q̇ was continuously measured by six-lead transthoracic impedance cardiography (PhysioFlow Enduro, Manatec Biomedical, Paris, France) and calculated as stroke volume (SV) × HR. Values are expressed as 60-s averages obtained at the end of a 3 min rest period. Due to logistical constraints, NIRS measurements were not performed at 1 h post-arrival at high-altitude (on day 0).

Prior to this three-minute resting period, 70 μl of capillary blood was obtained from a hyperaemic earlobe (pretreated with a topical heating cream; Capsolin, SIT s.r.l., Mede, Italy) via capillary tube (safeCLINITUBE; Radiometer Medical ApS, Copenhagen, Denmark). Immediately after collection, samples were mixed for 10 s and subsequently analyzed for acid–base status (pH, p50 and bicarbonate concentration ([HCO_3_^−^])), partial pressures of oxygen (pO_2_) and carbon dioxide (pCO_2_), oxygen saturation (SaO_2_), and hemoglobin concentration ([Hb]) using an ABL90 FLEX blood gas analyzer (Radiometer Medical ApS, Copenhagen, Denmark).

### Venous blood samples and analyses

Venous blood samples were collected at SL, within ~1 h post-arrival at altitude, as well as at the end of the morning physiological responses subsections. Samples were drawn from the antecubital vein (Becton Dickinson (BD) Vacutainer Safety-Lok, BD Biosciences, New Jersey, US) and collected into vacuum tubes containing either EDTA or silica clot activator (Becton Dickinson (BD) Vacutainer). Tubes were centrifuged (1500 rpm for 15 min at 4 °C), and the supernatant was stored at − 80 °C (EDTA plasma) or − 20 °C (serum) until later analysis. Commercially available enzyme-linked immunosorbent assays (ELISAs) were performed to determine [GDF15] (DY957, R&D, Minneapolis, MN) and [EPO] (DY286-05) in serum.

Plasma samples were further analysed to determine concentrations of advanced oxidation protein products ([AOPP]) and superoxide dismutase (SOD) activity. Plasmatic [AOPP] was quantified via spectrophotometry by measuring absorbance at 340 nm in a 96-well plate, following the protocol described by Taylor et al*.* (Taylor et al. 2015). AOPP levels were calculated against a chloramine-T standard curve (0–100 μM; 300 μL), which exhibits absorbance at 340 nm in the presence of potassium iodide. SOD activity was assessed according to the procedures described (Manferdelli et al. 2023; Debevec et al. 2015). Briefly, SOD activity was determined by subtracting the rate of blue formazan formation in PBS (blank) from that observed in plasma samples (the highest slope – sample slope). Absorbance was measured spectrophotometrically at 560 nm over a 5-min period using 96-well microplates. The difference in slopes was then divided by the absorption coefficient of blue formazan at 450 nm (0.019) and subsequently multiplied by the ratio of total well volume to sample volume (290/20).

### Sample size estimation

Sample size was determined a priori for the broader research project (G*Power version 3.1.9.7; Heinrich Heine University Düsseldorf, Germany). The power analysis was based on previously-reported effects of KE on hypoxia-induced changes in SpO_2_ during submaximal exercise (*η*_*p*_^*2*^=0.21; *d*≈0.52) (Poffe et al. 2021a). Assuming a group×time interaction in a mixed-effects ANOVA model (α=0.05, (1–β)=0.80), 26 participants (13 per group) were required. Seventeen participants per group completed the study. However, some data were missing or excluded because of insufficient physiological signal quality (NIRS TSI fit factor ≤95% or impedance cardiography signal quality ≤80%) or the development of symptoms unrelated to AMS (e.g., toothache, shivering, or mild muscle and joint pain). As a result, the final dataset included a minimum of 14 participants per group for all statistical comparisons.

### Statistical analyses

Statistical analyses were conducted in R (version 4.4.2, R Core Team, Vienna, Austria), whereas graphical visualizations were generated using GraphPad Prism (version 10.4.1, GraphPad Software, La Jolla, CA, USA). Separate two-way linear mixed-effects models were employed to examine differences between the IEK and PLA groups (main effect of group), time points (4 to 7 high-altitude time points), and to establish the group×time interaction. To adjust for potential between-group baseline differences at sea level (without supplementation) when estimating the main effect of group and the group×time interaction, the corresponding SL values were included as a covariate in all models. To account for inter-individual variability, participant ID was included as a random intercept in all models. When significant group×time interactions or main effects of time were observed, Šidák-adjusted *post-hoc* comparisons were performed to identify specific factor-level differences. Additionally, between-group differences in AMS incidence over time at high-altitude were evaluated using a mixed-effects logistic regression model, with group and time included as fixed effects and participant ID included as a random intercept. Unless otherwise specified, data are expressed as mean ± standard deviation (SD) and statistical significance defined as *P*<0.05. Where appropriate, effect sizes were reported as partial *η*^*2*^ (*η*_*p*_^*2*^) for main and interaction effects and Cohen's *d* for *post-hoc* pairwise comparisons. Cohen's *d* was interpreted as trivial (< 0.25), small (≥ 0.25), moderate (≥ 0.50), or large (≥ 1.00) (Cohen 1992).

## Results

### Baseline characteristics

Table 1 presents the baseline characteristics of the two groups.

**Table 1:** Baseline characteristics of participants in the IEK and PLA groups, including descriptive statistics (mean ± SD)

	**IEK group (*n* = 17)**	**PLA group (n = 17)**
Age (yr)	23 ± 2	24 ± 3
BMI (kg⋅m^−2^)	22.8 ± 2.0	22.3 ± 1.9
Body fat (%)	15 ± 4	15 ± 4
V̇O_2_peak (mL⋅kg^−1^⋅min^−1^)	53.5 ± 7.0	53.7 ± 5.3
Peak power output (W)	326 ± 65	318 ± 45
Blood hemoglobin concentration (g⋅L^−1^)	14.9 ± 1.7	15.0 ± 1.0
Hematocrit (%)	43 ± 4	45 ± 3
Forced vital capacity (L)	5.7 ± 1.0	5.5 ± 0.8
FEV1 (L)	4.6 ± 0.8	4.4 ± 0.7
Peak expiratory flow rate (L⋅s^−1^)	9.2 ± 2.2	9.7 ± 2.1

Peak oxygen uptake (V̇O_2_peak) and peak power output were determined during preliminary testing using a ramp-incremental exercise test protocol (2 min @ 50 W then + 1 W⋅3 s^−1^ until volitional exhaustion) performed on a cycle ergometer. *BMI* body mass index, *FEV1* forced expiratory volume in 1s, *IEK* intermittent exogenous ketosis, *PLA* placebo

### Blinding assessment

In both groups, 11 out of 17 participants (65%) correctly identified their group allocation. The mean confidence ratings were comparable between the IEK and PLA groups (67 ± 15% *vs*. 66 ± 16%, respectively).

### Capillary blood [β-HB]

KE ingestion robustly elevated capillary [β-HB] throughout the high-altitude exposure (group×time interaction *P*<0.001, *η*_*p*_^*2*^=0.39; IEK *vs*. PLA across 12 time points: all *P*<0.001, all *d*≥3.75), with average values at the individual high-altitude time points ranging from 1.9 to 3.9 mM in the IEK group (overall range 0.8–5.9 mM), compared with average values ≤0.4 mM in the PLA group (overall range 0.2–0.8 mM) (Fig. 1).

### Acute mountain sickness symptoms

AMS incidence (Table 2) peaked at 25–35% after 8 h at 3375 m, yet did not differ between IEK and PLA across the entire high-altitude exposure (overall 53% *vs*. 41%; time effect *P*=0.076, group effect *P*=0.469, group×time interaction *P*=0.822). Symptom severity increased at 8 h and 16 h relative to SL (both *P*<0.001, both *d*≥1.07), then decreased to match SL values from 32 h onwards, with no group×time interaction (*P*=0.896).

**Table 2:** Incidence of acute mountain sickness (AMS; number of cases and percentage) and AMS symptoms scores (median and interquartile range) across time points in the IEK and PLA groups

	IEK group		PLA group		IEK group	PLA group
Time point	AMS+ [*n* (%)]	AMS- [*n* (%)]	AMS+ [*n* (%)]	AMS- [*n* (%)]	AMS score [median (Q1–Q3)]	AMS score [median (Q1–Q3)]
SL	0 (0)	17 (100)	0 (0)	17 (100)	1 (0–1)	1 (0–1)
8 h (Day 0)	6 (35)	11 (65)	4 (25)	12 (75)	2 (1–3)^**#**^	2 (1–2)^**#**^
16 h (Day 1)	5 (29)	12 (71)	4 (24)	13 (76)	2 (1–3)^**#**^	1 (1–2)^**#**^
32 h (Day 1)	1 (6)	16 (94)	1 (6)	16 (94)	1 (0–2)	1 (0–1)
40 h (Day 2)	3 (18)	14 (82)	2 (12)	15 (88)	1 (0–2)	0 (0–1)
56 h (Day 2)	1 (6)	16 (94)	3 (18)	14 (82)	0 (0–2)	0 (0–1)
64 h (Day 3)	1 (6)	16 (94)	1 (6)	16 (94)	0 (0–2)	0 (0–1)
Overall	9 (53)	8 (47)	7 (41)	10 (59)	1 (0–2)	0 (0–1)

*AMS+* acute mountain sickness positive participants, *AMS-* acute mountain sickness negative participants,* n* number of cases, *Q1* first quartile, *Q3* third quartile. ^**#**^ *P*<0.001 *vs*. SL in both IEK and PLA groups

### Morning physiological responses

As noted in Table 3, SpO_2_ decreased from SL to 16 h post-arrival, and remained reduced through 64 h (all *P*<0.001 and *d*≥4.03 *vs*. SL). Increases in HR and MAP were observed, consistent with early acclimatization responses (all *P*<0.001 and *d*≥1.04 *vs*. SL). No group or group×time interaction effects were noted for SpO_2_ or HR (all *P*≥0.234). In contrast, MAP was consistently lower in IEK relative to PLA group across the three mornings at high-altitude (group effect *P*=0.009, *η*_*p*_^*2*^=0.21, all *d*≥1.16). Time-domain HRV indices (pNN50, RMSSD) decreased at 16 h post-arrival relative to SL (both *P*≤0.007 and *d*≥0.79). For pNN50, this reduction persisted up to 64 h post-arrival (all *P*<0.001 and *d*≥1.16), whereas RMSSD remained suppressed only up to 16 h (*P*=0.007, *d*=0.79) before returning to SL values at 40 h (*P*=0.059). No KE-related effects were observed across any HRV metric (all group effects or group×time interactions *P*≥0.184).

**Table 3:** Morning physiological responses across time points in the IEK and PLA groups

		IEK group	PLA group
SpO_2_ (%)	SL	99 ± 1	99 ± 1
	16 h (Day 1)	87 ± 3^#^	86 ± 4^#^
	40 h (Day 2)	88 ± 3^#^	87 ± 4^#^
	64 h (Day 3)	89 ± 3^#^	87 ± 4^#^
HR (bpm)	SL	56 ± 10	53 ± 9
	16 h (Day 1)	66 ± 13^#^	66 ± 12^#^
	40 h (Day 2)	66 ± 11^#^	69 ± 12^#^
	64 h (Day 3)	67 ± 18^#^	67 ± 11^#^
MAP (mmHg)	SL	86 ± 8	83 ± 7
	16 h (Day 1)	91 ± 8^#X*^	94 ± 8^#^
	40 h (Day 2)	93 ± 7^#X*^	97 ± 8^#^
	64 h (Day 3)	91 ± 7^#X*^	96 ± 6^#^
pNN50 (%)	SL	57 ± 20	54 ± 20
	16 h (Day 1)	38 ± 21^#^	37 ± 27^#^
	40 h (Day 2)	37 ± 19^#^	31 ± 29^#^
	64 h (Day 3)	34 ± 20^#^	41 ± 29^#^
RMSSD (ms)	SL	119 ± 68	114 ± 53
	16 h (Day 1)	71 ± 36^#^	83 ± 69^#^
	40 h (Day 2)	69 ± 36	105 ± 109
	64 h (Day 3)	68 ± 32	108 ± 90
LF (ms^2^)	SL	1212 ± 2183	1627 ± 1747
	16 h (Day 1)	670 ± 699	821 ± 713
	40 h (Day 2)	596 ± 683	987 ± 1997
	64 h (Day 3)	710 ± 824	1323 ± 2133
HF (ms^2^)	SL	1170 ± 1080	1005 ± 1041
	16 h (Day 1)	507 ± 530	947 ± 1348
	40 h (Day 2)	426 ± 379	914 ± 1585
	64 h (Day 3)	491 ± 625	815 ± 977

Data are mean ± SD. ^**#**^ *P*<0.05 *vs*. SL in both IEK and PLA groups, ^X^ group effect (*P*<0.05 IEK *vs*. PLA overall), * *P*<0.05 *vs*. PLA at the indicated time point

### Daytime physiological responses

**Fig. 2:**
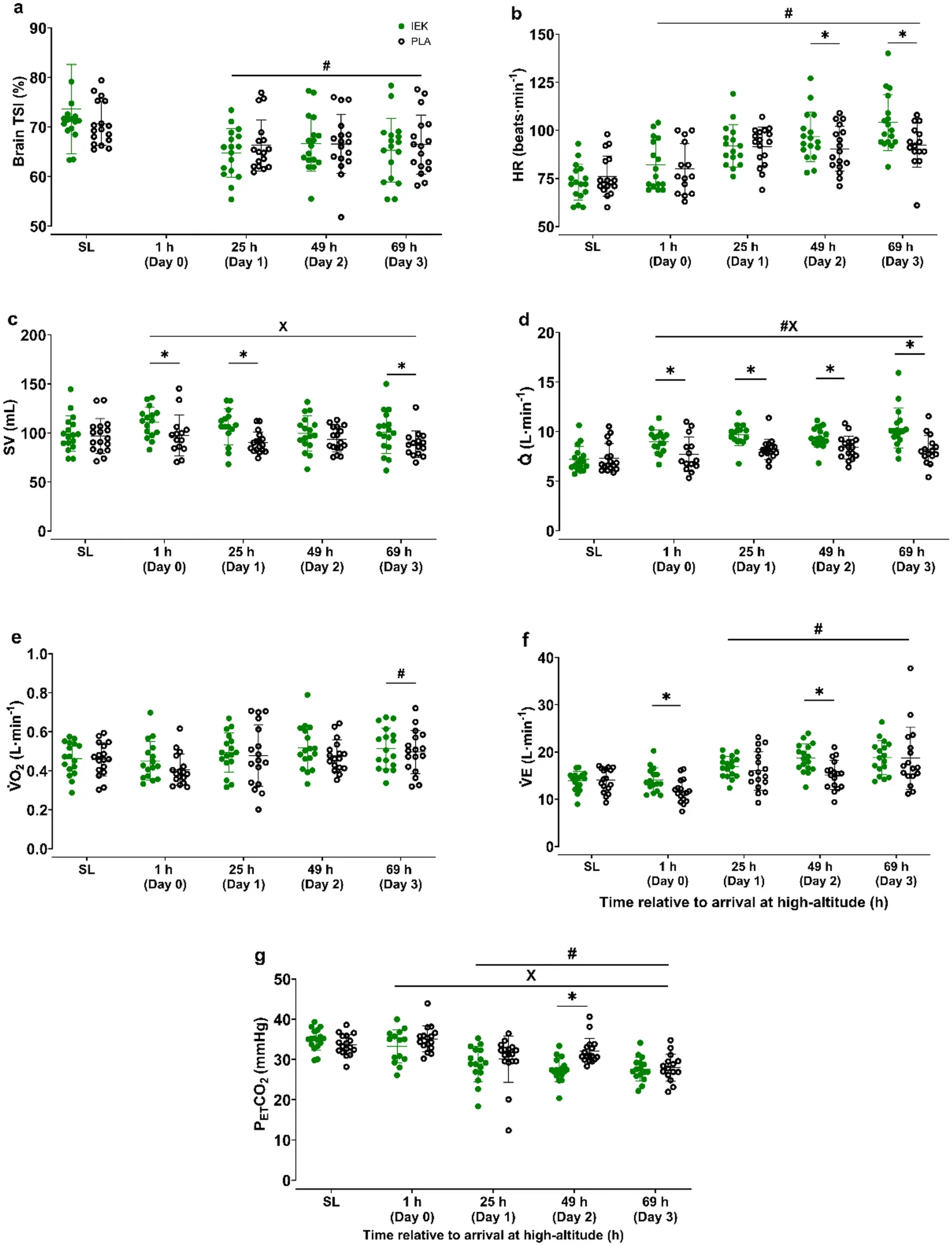
Daytime cerebral TSI (Brain TSI, **a**), heart rate (HR, **b**), stroke volume (SV, **c**), cardiac output (Q̇, **d**), oxygen uptake (V̇O_2_, **e**), minute ventilation (V̇E, **f**) and end-tidal partial pressure of carbon dioxide (P_ET_CO_2_, **g**) in participants assigned to the intermittent exogenous ketosis (IEK, green) and placebo (PLA, black) groups. All data are presented as individual values (solid and open circles) with group means (horizontal lines) ± SD (whiskers). Measurements were obtained without treatment at sea level (SL) and during a four-day high-altitude sojourn at 3375 m (Day 0–Day 3). ^#^ *P*<0.001 *vs*. SL, ^X^ group effect (*P*<0.05 IEK *vs*. PLA overall), * *P*<0.05 *vs*. PLA at the indicated time point

Brain TSI decreased from SL to 25 h post-arrival (*P*<0.001, *d*=1.22), and remained reduced throughout the high-altitude exposure (time effect *P*<0.001, *η*_*p*_^*2*^=0.26, *d*≥1.02; Fig. 2a).

Daytime HR increased from SL upon arrival at 3375 m and remained elevated throughout the exposure (time effect *P*<0.001, *η*_*p*_^*2*^=0.70, *d*≥0.94), with higher HR in the IEK *vs*. PLA at 49 h and 69 h post-arrival (group×time interaction *P*=0.024, *η*_*p*_^*2*^=0.10; *P*≤0.006 and* d*≥1.26 for IEK *vs*. PLA at 49 h and 69 h, respectively; Fig. 2b). Overall, SV remained comparable to SL across four days at 3375 (all *P*≥0.062 *vs*. SL), and was consistently higher in the IEK *vs*. PLA group across all daytime asessments at high-altitude (group effect *P*=0.012, *η*_*p*_^*2*^=0.19, all *d*≥0.53; Fig. 2c). As a result, Q̇ was elevated relative to SL throughout the entire high-altitude exposure (time effect *P*<0.001, *η*_*p*_^*2*^=0.10; all *P*<0.001 *vs*. SL, all* d*≥1.12), and was consistently higher in IEK *vs*. PLA at high-altitude (group effect *P*<0.001, *η*_*p*_^*2*^=0.50, all* d*≥1.10; Fig. 2d).

V̇O_2_ remained comparable to SL until 49 h post-arrival (all *P*≥0.098), then increased at 69 h (*P*=0.024,* d*=0.68; Fig. 2e). Overall, V̇E was unchanged from SL to 1 h after arrival at altitude (*P*=0.345), but rose at all later time points (all *P*≤0.003 *vs*. SL, all *d*≥1.00; time effect *P*<0.001, *η*_*p*_^*2*^=0.48), with higher V̇E in IEK *vs*. PLA at 1 h and 49 h post-arrival (group×time interaction *P*=0.037, *η*_*p*_^*2*^=0.08; *P*≤0.029 and *d*≥0.98 for IEK *vs*. PLA at 1 h and 49 h, respectively; Fig. 2f). In parallel, P_ET_CO_2_ remained stable from SL to 1 h post-arrival (*P*>0.999), then declined by 25 h (*P*<0.001, *d*=1.54) and remained lowered throughout the remainder of the four-day sojourn (all *P*<0.001 and* d*≥1.48 *vs*. SL; time effect *P*<0.001, *η*_*p*_^*2*^=0.49; Fig. 2g). In addition, P_ET_CO_2_ was consistently lower in IEK *vs*. PLA at high-altitude (group effect *P*=0.028, *η*_*p*_^*2*^=0.25, all* d*≥0.30).

### Capillary blood gas and acid–base

Table 4 summarises capillary blood gas and acid–base responses. High-altitude exposure reduced pO_2_ and SaO_2_ relative to SL (time effects *P*<0.001 and *η*_*p*_^*2*^≥0.85 for pO_2_ and SaO_2_, respectively). SaO_2_ showed no group (*P*=0.414) or group×time interaction (*P*=0.561), whereas pO_2_ was consistently higher in IEK *vs*. PLA at high-altitude (group effect *P*=0.049, *η*_*p*_^*2*^=0.15, all *d*≥0.39). p50 showed a group×time interaction (*P*=0.043, *η*_*p*_^*2*^=0.11), but *post-hoc* analysis revealed no IEK *vs*. PLA differences at any high-altitude time point (all *P*≥0.078). Blood pH showed no effect of time (*P*=0.262) or group×time interaction (*P*=0.358) but a significant group effect was present (*P*<0.001, *η*_*p*_^*2*^=0.48), indicating consistently lower pH in IEK *vs*. PLA at high-altitude (all *d*≥1.08).

**Table 4:** Capillary blood gasses, acid–base balance and total hemoglobin concentration across time points in the IEK and PLA groups

		IEK	PLA
pH	SL	7.416 ± 0.014	7.432 ± 0.054
	1 h (Day 0)	7.414 ± 0.033^X^*	7.451 ± 0.026
	25 h (Day 1)	7.422 ± 0.015^X^*	7.450 ± 0.015
	49 h (Day 2)	7.406 ± 0.017^X^*	7.446 ± 0.025
	69 h (Day 3)	7.418 ± 0.014^X^*	7.446 ± 0.027
pCO_2_ (mmHg)	SL	39.5 ± 2.4	37.4 ± 6.0
	1 h (Day 0)	32.0 ± 5.3^#X^*	34.5 ± 2.3^#^
	25 h (Day 1)	29.4 ± 3.1^#X^*	31.7 ± 1.9^#^
	49 h (Day 2)	28.0 ± 3.5^#X^*	31.5 ± 2.4^#^
	69 h (Day 3)	27.8 ± 3.3^#X^*	29.8 ± 2.2^#^
pO_2_ (mmHg)	SL	86.2 ± 4.3*	96.7 ± 18.3
	1 h (Day 0)	59.5 ± 12.2^#X*^	54.7 ± 9.9^#^
	25 h (Day 1)	55.6 ± 4.2^#X^	53.7 ± 3.1^#^
	49 h (Day 2)	58.2 ± 3.3^#X^	54.8 ± 3.5^#^
	69 h (Day 3)	56.7 ± 3.9^#X^	54.2 ± 4.0^#^
SaO_2_ (%)	SL	97.2 ± 0.65	97.6 ± 1.03
	1 h (Day 0)	88.9 ± 2.24^#^	88.7 ± 1.93^#^
	25 h (Day 1)	89.4 ± 2.87^#^	89.2 ± 1.96^#^
	49 h (Day 2)	90.3 ± 1.91^#^	89.4 ± 2.03^#^
	69 h (Day 3)	90.1 ± 2.25^#^	89.1 ± 2.15^#^
p50 (mmHg)	SL	25.79 ± 1.21	25.27 ± 1.62
	1 h (Day 0)	25.73 ± 1.39	24.56 ± 1.18
	25 h (Day 1)	25.39 ± 1.53	25.02 ± 0.89
	49 h (Day 2)	25.74 ± 1.32	25.18 ± 1.43
	69 h (Day 3)	25.26 ± 1.21	25.18 ± 1.07
[HCO_3_^−^] (mM·L^−1^)	SL	25.3 ± 1.4	24.7 ± 1.7
	1 h (Day 0)	20.9 ± 1.9^#X^*	24.1 ± 1.0^#^
	25 h (Day 1)	19.1 ± 2.0^#X^*	22.0 ± 1.1^#^
	49 h (Day 2)	17.6 ± 2.4^#X^*	21.6 ± 1.0^#^
	69 h (Day 3)	18.0 ± 2.1^#X^*	20.5 ± 0.9^#^
[Hb] (g·dL^−1^)	SL	15.8 ± 1.4	15.5 ± 1.1
	1 h (Day 0)	17.0 ± 1.9^#^	16.2 ± 1.3^#^
	25 h (Day 1)	16.3 ± 1.5^#^	16.5 ± 0.8^#^
	49 h (Day 2)	17.1 ± 1.7^#^	17.1 ± 1.2^#^
	69 h (Day 3)	17.7 ± 1.8^#^	17.6 ± 0.9^#^

Data is mean ± SD.^**#**^ *P*<0.05 *vs*. SL in both IEK and PLA groups, ^X^ group effect (*P*<0.05 IEK *vs*. PLA overall), * *P*<0.05 *vs*. PLA at the indicated time point

Overall, pCO_2_ decreased from SL to 1 h post-arrival, and remained reduced throughout the high-altitude exposure (all *P*<0.001 and *d*≥2.16 *vs*. SL), with consistently lower values in IEK *vs*. PLA (group effect *P*=0.002, *η*_*p*_^*2*^=0.34, all* d*≥1.31). Similarly, [HCO_3_^−^] decreased upon arrival and remained reduced *vs*. SL (all *P*<0.001 and *d*≥2.70 *vs*. SL), with consistently lower values in IEK *vs*. PLA (group×time interaction *P*<0.001, *η*_*p*_^*2*^=0.24; all *d*≥2.66). [Hb] increased from SL upon arrival (*P*<0.001, *d*≥1.13) and remained elevated over the subsequent three days at 3375 m (time effect *P*<0.001, *η*_*p*_^*2*^=0.51, all *d*≥1.13; group effect *P*=0.921; group×time interaction *P*=0.635).

### Hematological markers

**Fig. 3:**
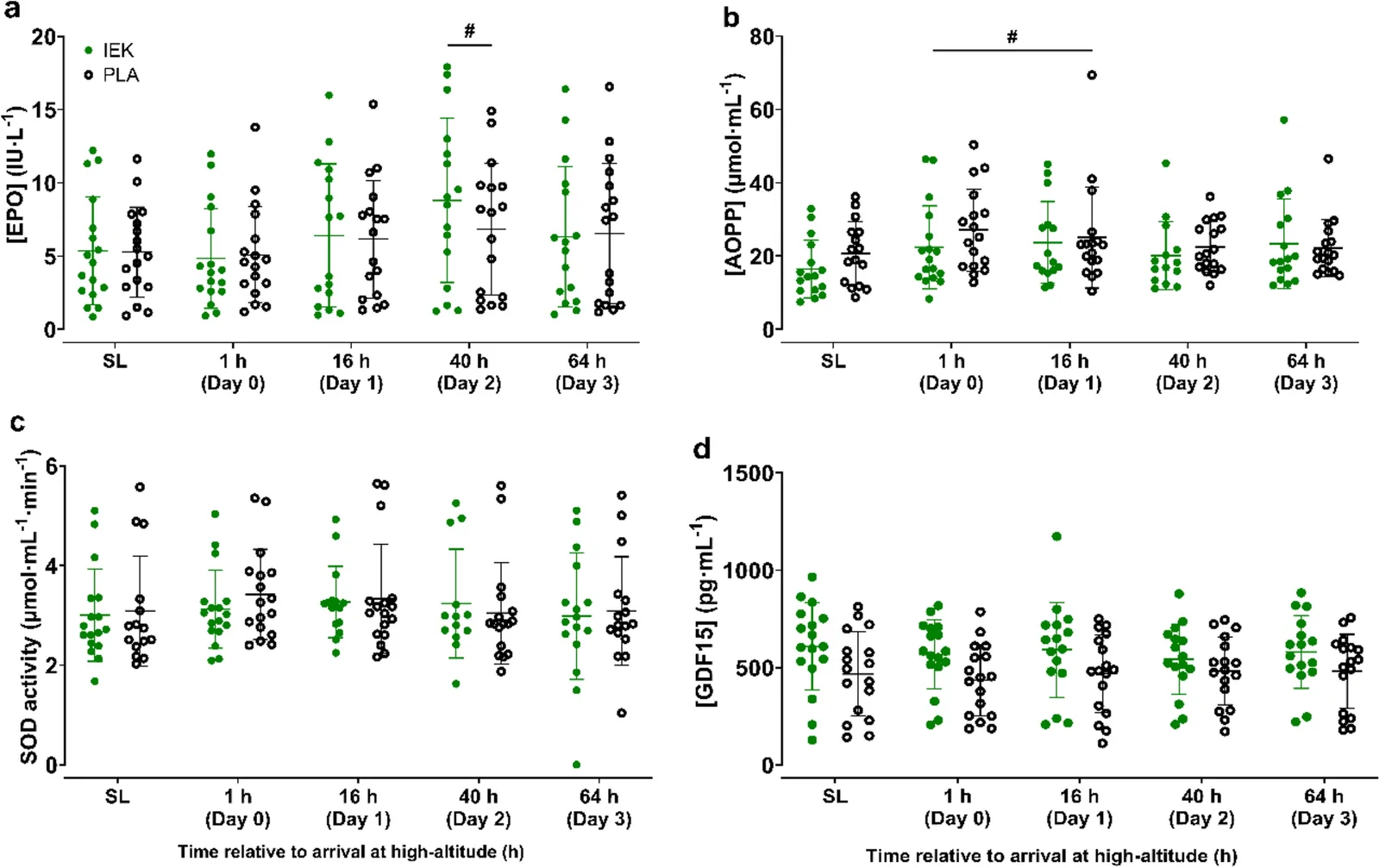
Serum concentrations of erythropoietin ([EPO], **a**), plasma levels of advanced oxidation protein products ([AOPP], **b**), and superoxide dismutase activity (SOD activity, **c**) and serum growth differentiation factor 15 concentration ([GDF15], **d**) in participants allocated to the intermittent exogenous ketosis (IEK, green closed circles) and placebo (PLA, black closed circles) groups. All data are presented as individual values (solid and open circles) with group means (horizontal lines) ± SD (whiskers). Measurements were obtained without treatment at sea level (SL) and during a four-day high-altitude sojourn at 3375 m (Day 0–Day 3). ^#^*P*<0.001 *vs*. SL in both IEK and PLA groups

Serum [EPO] increased transiently during high-altitude exposure (time effect *P*<0.001, *η*_*p*_^*2*^=0.17), with a difference relative to SL noted only at 40 h (*P*<0.001, *d*=0.98; Fig. 3a). No group (*P*=0.571) or group×time interaction (*P*=0.301) effects were observed. Plasma [AOPP] exhibited a moderate early increase at 1 h (*P*=0.002, *d*=0.88) and 16 h post-arrival (*P*=0.004, *d*=0.85), before returning to SL values (Fig. 3b), while SOD activity remained unchanged throughout the high-altitude sojourn (all *P*≥0.072 *vs*. SL; Fig. 3c). No group-related effects were observed for either marker. Serum [GDF15] displayed neither group×time interaction (*P*=0.099), nor main effect of time (*P*=0.239) or group (*P*=0.485; Fig. 3d).

## Discussion

We sought to investigate whether intermittent exogenous ketosis can attenuate AMS and modulate key physiological responses during early acclimatization to terrestrial high-altitude (3375 m). Although KE ingestion consistently elevated circulating [β-HB] during the day and before sleep at altitude, this did not translate into reduced AMS incidence nor severity. KE ingestion induced (keto)acidosis, resulting in augmented ventilatory response, yet also failed to mitigate hypoxia-induced blood or cerebral deoxygenation. Moreover, KE ingestion increased daytime cardiac output and lowered morning blood pressure, but did not meaningfully affect autonomic, eryrthropoietic, or redox responses.

Previous evidence showed that exogenously induced ketosis improved hypoxic tolerance and reduced self-reported AMS symptoms after 4 h of exposure to simulated high-altitude (4000–4500 m) (Stalmans et al. 2024). However, because these symptoms are non-specific and AMS typically develops after ~6–10 h of hypoxic exposure (Hackett and Roach 2001), these early symptoms should be interpreted with caution. In contrast, the present study did not corroborate these findings under hypobaric hypoxic conditions during a four-day high-altitude sojourn. Several factors may explain discrepancies with these previous findings, most notably the difference in hypoxic severity (4000–4500 m (Stalmans et al. 2024) *vs*. 3375 m), which likely played a greater role than the duration of hypoxic exposure. This interpretation is supported by the higher incidence of AMS after only 4 h at ~4000 m (64%) (Stalmans et al. 2024) compared with the maximum incidence observed over 64 h at 3375 m in the present study (35%). Differences in physical activity (one maximal and one submaximal exercise bout (Stalmans et al. 2024) *vs*. one submaximal exercise bout before peak AMS symptoms severity), may have further contributed to these discrepant findings. Furthermore, as this was the first study to assess the effects of KE on AMS at terrestrial altitude, the hypobaric hypoxic stimulus (Ferrarini et al. 2026), together with other environmental factors (*e.g.,* cold temperatures, dry air, and nutritional challenges) (Millet and Debevec 2020) and high degree of inter-individual variability in susceptibility to AMS (Luks et al. 2017) may have limited the ability of our parallel-group design to detect KE-related effects.

The early onset of AMS is closely associated with a hypoxia-induced drop in blood pO_2_ and consequent SpO_2_ reduction (Burtscher et al. 2004). KE administration has been shown to attenuate this drop in SpO_2_ upon exposures to simulated altitudes (~ 2500–6100 m) under normobaric hypoxia (Stalmans et al. 2024; Stalmans et al. 2025b; Poffe et al. 2021a; McClure et al. 2024a; McClure et al. 2024b), as well as following ~45 h of exposure at terrestrial high-altitude (Narang et al. 2025). These effects have been attributed to the KE-induced metabolic (keto-)acidosis and subsequent augmented V̇E response, resulting in a proportionally greater increase in (capillary) blood pO_2_ relative to the rightward shift of the oxyhemoglobin dissociation curve (ODC; increase in p50), thereby yielding a net SpO_2_ increase (Stalmans et al. 2025a). However, these effects have not been consistently observed (Stalmans et al. 2025a; Stalmans et al. 2025b; Tominec et al. 2026, 2025), with no between-group differences in morning awakening SpO_2_ or daytime SaO_2_ also noted in this study. It has recently been proposed (Stalmans et al. 2025a; Thiessen et al. 2025) that the efficiency of exogenous ketosis in increasing SpO_2_ and thereby potentially mitigating AMS symptoms increases in conditions when SpO_2_ is positioned on the “steep” portion of the ODC. Indeed, KE ingestion across four days at 3375 m in our study increased V̇E and pO_2_ (without altering p50), yet SpO_2_ and SaO_2_ consistently remained positioned on the “flatter” ODC segment (pO_2_ >25 mmHg above p50 (Collins et al. 2015)), which likely explains the absence of SpO_2_ and AMS symptoms improvement.

KE ingestion has also been shown to increase cerebral oxygenation (Stalmans et al. 2024; Stalmans et al. 2025a; Vermaerke et al. 2026) in normobaric hypoxia, while reducing cerebral blood flow (Stalmans et al. 2024). This could possibly reflect lower oxygen extraction (Stalmans et al. 2024) due to greater energetic efficiency (Suissa et al. 2021) and preferential utilization of ketone bodies over glucose (Kirsch and D'Alecy 1984). Nevertheless, these mechanistic interactions require further systematic investigation, since this study did not reveal between-group differences in cerebral oxygenation at rest under hypobaric hypoxia. However, we contend that the observed discrepancies are likely attributable to the moderate hypoxic stimulus employed, as more pronounced physiological perturbations typically emerge at altitudes ≥4000 m (Richalet et al. 2012). In addition, responses may differ between hypobaric and normobaric hypoxia, as hypobaria per se independently alters ventilatory control and oxygenation (Ferrarini et al. 2026). Specifically, under hypobaric relative to normobaric hypoxia, reduced air density and altered gas diffusivity are presumed to enhance ventilatory efficiency (Ferrarini et al. 2026) and promote greater respiratory alkalosis (Cerretelli and Samaja 2003), thereby inducing a (leftward) shift of the ODC and altering processes throughout the oxygen transport cascade. Alternatively, the absence of detectable effects may reflect limited statistical power (*n* = 17 per group) and the increased number of repeated parallel-group measurements (4–5 timepoints), potentially obscuring KE-related effects in this *vs*. our previous study (Narang et al. 2025).

Increased [β-HB] has also been shown to enhance myocardial contractility and/or vasodilation (Selvaraj et al. 2022; Nielsen et al. 2019; Gopalasingam et al. 2024), enhance sympathoexcitation (Stalmans et al. 2024; Stalmans et al. 2025b), and act as an antidiuretic (Poffé et al. 2020; Robberechts et al. 2022; Poffe et al. 2021a), which may influence AMS susceptibility through cardiovascular response modulation (Bruno et al. 2015). Indeed, KE ingestion increased daytime HR at 49 and 69 h post-arrival and consistently elevated SV, resulting in increased Q̇ throughout the four-day sojourn. These findings align with prior evidence of acute increases in resting Q̇ and SV in normoxia (Selvaraj et al. 2022; Nielsen et al. 2019; Gopalasingam et al. 2024) and are consistent with recent observations during moderate-intensity exercise in the early phase of high-altitude acclimatization within the same project (Tominec et al. 2026). Although the mechanisms underlying these improvements are not fully understood, the present findings suggest a multifactorial basis. These likely include KE-induced increases in SV, potentially mediated by enhancements in sarcomere shortening (Klos et al. 2019), systemic vasodilation (Walsh et al. 2021; McCarthy et al. 2021), and/or attenuated diuresis (Poffé et al. 2020; Robberechts et al. 2022; Poffe et al. 2021b), alongside elevated HR driven by KE-induced augmented sympathetic activation (Robberechts et al. 2022; Stalmans et al. 2024; Stalmans et al. 2025b; Robberechts et al. 2023; Selvaraj et al. 2022; Nielsen et al. 2019). Nonetheless, it is important to note that, in the present work, these cardiovascular changes did not translate into attenuation of AMS development.

Furthermore, HRV analysis represents a valuable tool for assessing autonomic (im)balance (Tanner et al. 2021), which has also been implicated in AMS development (Berger et al. 2020). We previously demonstrated a greater attenuation of HRV indices with KE ingestion both during the initial 4 h at a simulated altitude of 4000 m (Stalmans et al. 2024), and after 2 h and 13 h at 3000 m (Stalmans et al. 2025b), collectively suggesting a more pronounced shift toward sympathetic dominance when participants were in exogenous ketosis. In the present study, no such effects were observed, which instead aligns with the HRV and baroreflex findings reported from the ~45 h time point within the same project (Narang et al. 2025). The sources of these discrepancies remain unclear, indicating that KE-mediated modulation of autonomic function may depend on hypoxia type (hypobaric *vs*. normobaric) and/or exposure duration. However, comparative analyses across assessment conditions in the current study (morning, post-awakening without KE *vs*. midday (pre-exercise) following KE ingestion) indicate that the observed KE effects were limited to ‘daytime’ measurements (~ 30 min post single KE bolus ingestion). This finding suggests that augmented physiological responses during early altitude acclimatization primarily depend on the acute presence of exogenous ketosis, as these responses are potentially transient and supplementation-driven, rather than sustained under prolonged intermittent intake. Indeed, in the current study, IEK *vs*. PLA demonstrated elevated HR during ‘daytime’ assessments, whereas HRV indices and ‘post-awakening’ HR remained similar between the groups, which may have contributed to the observed discrepancies.

In contrast, MAP upon awakening at high-altitude was consistently lower in the IEK *vs*. PLA group, which diverges from sea level findings where KE exerted no measurable effect on blood pressure (Perissiou et al. 2025; Seto et al. 2025). These data, suggest that only specific physiological systems—particularly blood pressure regulation—may be modulated by prolonged KE supplementation under (prolonged) hypoxic conditions. The observed reduction in MAP (even in the absence of acute ketosis at the time of the measurement) in hypoxia may be attributable to previously reported β-HB-mediated vasodilatory effects (McCarthy et al. 2019, 2021) and improved vascular function (Walsh et al. 2021). In addition, ketones have been suggested to induce antidiuretic effects (Poffé et al. 2020; Robberechts et al. 2022; Poffe et al. 2021a), which could promote fluid retention and increase circulating plasma volume while still being compatible with the lower MAP observed in the IEK group. However, the potential contribution of this mechanism, and the broader cardiovascular effects of exogenous ketone supplementation during (prolonged) hypoxic exposure, remain to be established.

Previous work has also shown that, both acute post-exercise KE ingestion (Evans et al. 2023) and daily KE administration during a 3-week overreaching period (Poffe et al. 2023) can augment serum [EPO] by ~20–26% compared with a PLA condition. However, although a ~47% increase in serum [EPO] relative to SL was observed after 40 h at 3375 m in this study, KE supplementation had no independent effect. This finding is consistent with observations from our previous investigation conducted after 5 h of exposure to simulated high-altitude (4000 m) (Stalmans et al. 2025a), in which normobaric hypoxia markedly increased circulating [EPO] (~ 56%), irrespective of (more frequent) KE supplementation. With SpO_2_ seemingly unaffected in this study, it seems that the hypoxic stimulus itself likely dominated KE-induced [EPO] regulation in these conditions.

High-altitude is known to elevate oxidative stress and reduce antioxidant capacity (Debevec et al. 2017; Faiss et al. 2013), both considered relevant to AMS pathogenesis (Pena et al. 2022). Our study demonstrated a ~33% increase in [AOPP] across 16 h at high-altitude compared to SL, whereas SOD activity remained unchanged, regardless of KE *vs*. placebo supplementation. KE administration has been hypothesized to attenuate oxidative stress, based on inherent antioxidant properties of β-HB and the upregulation of antioxidant enzymes (Rojas-Morales et al. 2020). Yet limited evidence from two sea level studies investigating the effects of exogenous ketones on redox balance similarly shows no impact on exercise-induced oxidative stress, inflammation, or antioxidant capacity (McAllister et al. 2019; Waldman et al. 2022). Interestingly, KE supplementation has also been shown to blunt the progressive rise in the stress-sensitive hormone [GDF15] during training overload (Poffe et al. 2019), as well as during exercise following 2 h of exposure at simulated altitude (3000 m) (Poffe et al. 2021a). However, serum [GDF15] was not altered under conditions of exogenous ketosis in the present study. Although the mechanisms underlying KE-related modulation of stress-sensitive hormonal responses are not fully elucidated, our findings may partly stem from previously reported KE-induced concomitant reductions in blood glucose concentration. Such decreases could provoke a compensatory increase in glucocorticoid secretion, thereby masking any direct physiological effects of KE ingestion (Waldman et al. 2022).

### Methodological considerations & limitations

As with any field-based high-altitude study, some limitations should be acknowledged. Firstly, practical and logistical constraints during the high-altitude phase precluded the implementation of rigorous dietary standardisation or continuous surveillance of participants’ nutritional intake. In addition, daytime testing was not performed under fasted conditions or following fully-standardized pre-measurement meals, which may have contributed to inter-individual variability in substrate availability and, consequently, to differences in the physiological responses elicited by KE ingestion. Furthermore, the KE and placebo beverages were not isocaloric. Although this difference cannot be entirely excluded as a potential confounder, its influence was likely limited, as all participants, regardless of group assignment, received three daily meals of similar composition and portion size served at relatively consistent times according to the mountain hut schedule, with additional food available if required. Finally, all participants were instructed to maintain their habitual dietary practices and to abstain from the use of nutritional supplements for at least two months prior to, and throughout, the duration of the study. Moreover, any ventilatory effects of KE are more likely attributable to bicarbonate buffering than to oxidative metabolism of the ingested KE (Prins et al. 2021; Dorner et al. 2024). Although recruitment was inclusive of both biological sexes, women comprised only a small proportion of the sample (six participants; 18%), and menstrual cycle phase was not controlled. While this may limit the generalisability of the findings, recent evidence suggests that menstrual cycle phase has a negligible impact on the physiological responses examined in this study (Tagliapietra et al. 2024, 2025; Citherlet et al. 2024).

## Conclusion

The present results suggest that although KE ingestion effectively induced intermittent exogenous ketosis during a four-day sojourn at a terrestrial altitude of 3375 m, this did not reduce AMS incidence and severity. KE intake altered hypoxia-related ventilatory, cardiac, and acid–base responses during daytime, yet did not improve blood or cerebral oxygenation, potentially explaining the unchanged AMS trajectory. Moreover, our data also suggest that KE intake effects are potentially time- and system-specific: autonomic regulation appears acutely dependent on the presence of exogenous ketosis, whereas vascular regulation may be modulated by longer, regular KE administration. Prospective studies investigating the efficiency of intermittent exogenous ketosis to facilitate altitude acclimatization employing prolonged exposures to different altitudes combined with and without exercise are warranted.

## Data Availability

The datasets generated during and/or analyzed during the current study are available in the Repository of the University of Ljubljana, https://repozitorij.uni-lj.si/IzpisGradiva.php?id=182484&lang=eng

## Abbreviations

AMS: Acute mountain sickness
AOPP: Advanced oxidation protein products
ARR: Arrival
BMI: Body mass index
DBP: Diastolic blood pressure
EPO: Erythropoietin
FEV1: Forced expiratory volume in 1 s
GDF15: Growth differentiation factor 15
Hb: Hemoglobin
HF: Power of the high-frequency band
HR: Heart rate
HRV: Heart rate variability
IEK: Intermittent exogenous ketosis
KE: Ketone monoester
LF: Power of the low-frequency band
MAP: Mean arterial pressure
pCO_2_: Partial pressure of carbon dioxide
P_ET_CO_2_: End-tidal partial pressure of carbon dioxide
pNN50: Percentage of adjacent NN intervals that differ by more than 50 ms
pO_2_: Partial pressure of oxygen
PLA: Placebo
Q̇: Cardiac output
RMSSD: The root mean square of successive differences
SaO_2_: Arterial blood oxygen saturation
SBP: Systolic blood pressure
SL: Sea level
SOD: Superoxide dismutase
SpO_2_: Peripheral blood oxygen saturation
SV: Stroke volume
TSI: Tissue saturation index
V̇CO_2_: Carbon dioxide production
V̇E: Minute ventilation
V̇O_2_: Oxygen uptake
V̇O_2_peak: Peak oxygen uptake
β-HB: β-Hydroxybutyrate
